# Deficiency in p53 is required for doxorubicin induced transcriptional activation of NF-κB target genes in human breast cancer

**DOI:** 10.18632/oncotarget.1556

**Published:** 2013-11-23

**Authors:** Alba Dalmases, Irene González, Silvia Menendez, Oriol Arpí, Josep Maria Corominas, Sonia Servitja, Ignasi Tusquets, Cristina Chamizo, Raúl Rincón, Lluis Espinosa, Anna Bigas, Pilar Eroles, Jessica Furriol, Anna Lluch, Ana Rovira, Joan Albanell, Federico Rojo

**Affiliations:** ^1^ Cancer Research Program, IMIM (Hospital del Mar Research Institute), Barcelona, Spain;; ^2^ Medical Oncology Department, Hospital del Mar, Barcelona, Spain;; ^3^ Pathology Department, Hospital del Mar, Barcelona, Spain;; ^4^ Autonomous University of Barcelona, Spain;; ^5^ Pathology Department, IIS-Fundación Jiménez Díaz, Madrid, Spain;; ^6^ Oncology and Hematology Department, Hospital Clinico Universitario, Valencia, Spain;; ^7^ Valencia Central University, Spain;; ^8^ Universitat Pompeu Fabra, Barcelona, Spain

**Keywords:** breast cancer, chemoresistance, NF-κB, p53, prognosis

## Abstract

NF-κB has been linked to doxorubicin resistance in breast cancer patients. NF-κB nuclear translocation and DNA binding in doxorubicin treated-breast cancer cells have been extensively examined; however its functional relevance at transcriptional level on NF-κB -dependent genes and the biological consequences are unclear. We studied NF-κB -dependent gene expression induced by doxorubicin in breast cancer cells and fresh human cancer specimens with different genetic backgrounds focusing on their p53 status.

NF-κB -dependent signature of doxorubicin was identified by gene expression microarrays in breast cancer cells treated with doxorubicin and the IKKβ-inhibitor MLN120B, and confirmed *ex vivo* in human cancer samples. The association with p53 was functionally validated. Finally, NF-κB activation and p53 status was determined in a cohort of breast cancer patients treated with adjuvant doxorubicin-based chemotherapy.

Doxorubicin treatment in the p53-mutated MDA-MB-231 cells resulted in NF NF-κB driven-gene transcription signature. Modulation of genes related with invasion, metastasis and chemoresistance *(ICAM-1, CXCL1, TNFAIP3, IL8)* were confirmed in additional doxorubicin-treated cell lines and fresh primary human breast tumors. In both systems, p53-defcient background correlated with the activation of the NF-κB -dependent signature. Furthermore, restoration of p53WT in the mutant p53 MDA-MB-231 cells impaired NF-κB driven transcription induced by doxorubicin. Moreover, a p53 deficient background and nuclear NF-κB /p65 in breast cancer patients correlated with reduced disease free-survival.

This study supports that p53 deficiency is necessary for a doxorubicin driven NF-κB -response that limits doxorubicin cytotoxicity in breast cancer and is linked to an aggressive clinical behavior.

## INTRODUCTION

Doxorubicin, an anthracycline commonly used to treat breast cancer, inhibits topoisomerase II enzyme, resulting in DNA damage that leads to apoptosis [1]. However, DNA damage induced by doxorubicin can also switch on the transcription factors p53 [2] and the nuclear factor kappa B (NF-κB), limiting drug cytotoxicity. NF-κB has five subunits: p65, p50, p52, c-Rel and RelB [3], that can form dimers. They are inactive in the cytoplasm in complex with inhibitory IκBs proteins. Once in the nucleus, NF-κB binds to the DNA κB sites, in the promoter or enhancer of its target genes. NF-κB dimers have distinct binding sites [4] and are modified post-translationally to recruit co-activators to activate transcription of genes participating in key cellular processes [5]. The combinatorial diversity of NF-κB dimers, postranslational modifications and association with transcriptional regulators contributes to the regulation of distinct, but sometimes overlapping sets of genes upon specific stimuli [6]. Two kinase subunits, IKKβ and IKKα, and a regulatory IKKγ (NEMO), constitute the IkappaB kinase complex (IKK) necessary for IκB phosphorylation and NF-κB activation. We have used in this study the IKKβ inhibitor MLN120B [7].

We have shown previously that doxorubicin activates NF-κB in BT474 breast cancer cells and pharmacological inhibition of NF-κB enhanced its antitumoral effects [8]. Similar results have been achieved by others [9, 10]. From a clinical perspective, we have showed that nuclear NF-κB /p65, as a surrogate marker of activation, correlated with resistance to anthracycline-based chemotherapy and worse outcome [11]. Furthermore, p65 activation was increased after neoadjuvant chemotherapy in breast cancer in residual disease [12]. Other studies have reported similar findings in breast cancer and other tumor types [13, 14].

It is now widely accepted that transcriptional induction of anti-apoptotic and prosurvival genes by NF-κB contributes to cancer cell chemoresistance, thus reinforcing the concept of targeting NF-κB in anticancer therapies [15]. However, recent reports have suggested that NF-κB activity in response to anthracyclines can also play pro-apoptotic functions. Specifically, daunorubicin suppresses the transcription of bcl-xL by NF-κB activation in osteosarcoma cells and doxorubicin represses NF-κB anti-apoptotic genes in breast cancer cells [16]. In this study, we aimed to gain further insight in NF-κB- regulated gene expression in response to doxorubicin treatment to clarify its functional importance in chemoresistance in breast cancer.

## RESULTS

### Doxorubicin induces the expression of metastasis related genes through NF-κB activation in MDA-MB-231 breast cancer cells

**Figure 1:**
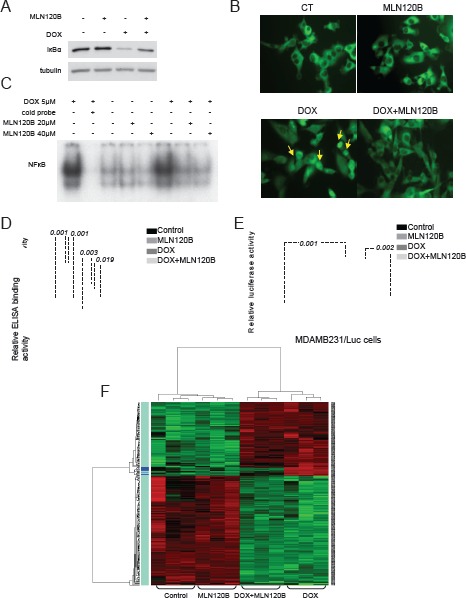
Doxorubicin activates the NF-κB and enhances the expression of migration, cell adhesion and metastasis related NF-κB target genes in MDA-MB-231 breast cancer cells Cells were cultured 90min in the presence or absence of MLN120B (20µM) and further incubated 4h with or without doxorubicin (5µM). Different steps of the NF-κB pathway were characterized. A, Levels of IκBα protein in whole protein lysates determined by Western Blot. α-tubulin served as the loading control. B, Representative images of p65 cellular distribution (nucleus/cytoplasm) determined by immunofuorescence (IF) using Alexa 488-coupled goat anti-rabbit IgG (green). Arrows indicate representative nuclear p65 signals in doxorubicin treated cells. C, DNA binding activities of NF-κB in nuclear extracts determined by EMSA. Lane 2 contains competitor “cold” NF-κB probe at 100-fold molar excess with the same nuclear extract than in Lane 1 as specificity control. D, DNA-binding activity of five NF-κB subunits analyzed in nuclear extracts using commercial ELISA kit. Bars represent the average of three independent experiments. Error bars represent standard deviations. E, MDA-MB-231 cells stably transfected with a NF-κB-luciferase reporter. To assay luciferase activity doxorubicin treatment was performed for 24h. Bars represent the average of three independent experiments. Error bars represent standard deviations. The results are reported as the percentage of fold increase in relative luminescence in arbitrary units (RLA) of treated sample using untreated condition as reference. F, Microarray gene expression profile was established in treated MDA-MB-231 cells. On the heat map, right panel indicates the genes differentially expressed comparing doxorubicin treated samples vs. samples treated with both MLN120B and doxorubicin. Green color indicates under expressed (down-regulated) genes; Red color indicates overexpressed (up regulated genes) and Black indicates no change.

Consistently with our previous findings in other cancer cells [8], doxorubicin induced IκBα degradation, p65 nuclear localization and increased NF-κB -DNA binding in the triple negative MDA-MB-231 cells. These effects were prevented by MLN120B, a specific inhibitor of the kinase IKKβ (Fig. 1A, 1B and 1C). NF-κB subunit composition was determined by ELISA-based assay of nuclear extracts showing a significant p65 and p50 binding to DNA induced by doxorubicin compared to control and these effects were prevented by MLN120B (Fig. 1D). The DNA-binding ability of the other NF-κB subunits was unaltered following doxorubicin treatment.

Previously published reports showed controversial data regarding the ability of doxorubicin-induced DNA-bound NF-κB to activate specific gene transcription. To clarify this issue, we used stably transfected MDA-MB-231 cells carrying a luciferase reporter gene under the control of NF-κB responsive elements (MDAMB231/kB-Luc cells). In these cells, doxorubicin exposure resulted in an increase of NF-κB regulated luciferase activity that was prevented by IKKβ inhibition (Fig. 1E). This result indicates that NF-κB induced by doxorubicin is overall transcriptionally active.

Next, we performed a microarray gene expression study to investigate the possibility that doxorubicin modulates the expression of specific NF-κB target genes. Upon 4 hours of doxorubicin exposure, more than 300 genes were differentially modulated in MDA-MB-231 compared with control cells (GEO accession number: #16789213) (Fig. 1F). Among them, a set of 12 genes was partially reverted to basal levels by adding MLN120B (Fig. 1F and Table S1). However, no significant differences were observed between MLN120B treated and control cells in the absence of doxorubicin.

### Transcriptional responses of doxorubicin-induced NF-κB activation varies among breast cancer cells

**Figure 2:**
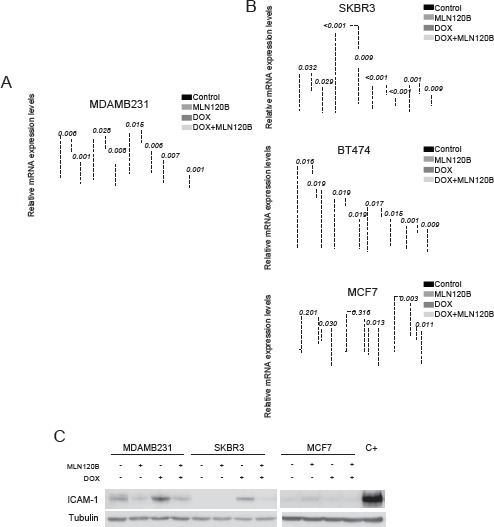
NF-κB -driven transcription by doxorubicin differs among breast cancer cells Four genes (*IL8*, *ICAM-1*, *CXCL-1* and *TNFAIP3*) were chosen for validation of the microarray results by qRT-PCR. Cells were treated as for the microarray experiment: A, MDA.MB-231 B, SKBR3, BT-474 and MCF-7. The relative gene expression level is normalized to the *RPLP0* gene. The graph shows the expression of each gene across different experimental conditions relative to their expression in control condition. C, ICAM-1 protein expression was determined in whole cell lysates of MDA-MB-231, SKBR3 and MCF-7 cells treated as for Fig 1e. α-tubulin served as the loading control. Cells treated with TNF-α were used as positive control.

Next, we independently tested by qRT-PCR four candidate genes selected from microarray data that were upregulated by doxorubicin and whose activation was prevented by MLN120B (*CXCL1*, *IL8*, *ICAM1* and *TNFAIP3*). These genes were selected because of their relevance in cell adhesion and migration (IL-8, *ICAM-1*), chemotherapy resistance (CXCL-1) and negative NF-κB regulation (*TNFAIP3*). In accordance with microarray data, the four genes were upregulated in doxorubicin- treated MDA-MB-231 cells and MLN120B pretreatment efficiently prevented their upregulation (Fig. 2A). Of note, in contrast with the array experiments where we did not detect expression changes in MLN120B treated cells compared with control, by qRT-PCR we observed significant differences between MLN120B treated and control samples in the absence of doxorubicin exposure (p=0.002 for *ICAM1*; p<0.001 for CXCL1; p=0.009 for TNFAIP3; p<0.001 for IL8). To determine whether the activation of NF-κB occurred in other phenotypically different breast cancer cell lines, we treated with doxorubicin the HER2-overexpressing SKBR3 and BT-474 and the ER-positive MCF-7 cells. Doxorubicin induced p65 nuclear translocation in all cell lines as shown by western blot and immunofluorescence (Supplementary Fig. S1A and S1B). In both BT-474 and SKBR3,doxorubicin induced NF-κB transcriptional activation as indicated by the upregulation of *IL8, ICAM1, CXCL1* and *TNFAIP3* genes that was prevented by MLN120B (Fig. 2B). However, gene expression induction of *IL8, ICAM1*, and *TNFAIP3* by doxorubicin was not observed in MCF-7 cells. *CXCL1* gene was not detectable at basal conditions and was excluded in the analysis. We then analyzed the modulation at protein level of *ICAM-1*. An increase of *ICAM-1* expression in MDA-MB-231 and SKBR3 cells under doxorubicin treatment, which was prevented by IKKβ inhibition, was demonstrated by western blot (Fig. 2C), although results on *ICAM-1* in BT-474 were non-conclusive (data not shown). According with the gene expression results, *ICAM-1* protein levels did not increase after doxorubicin exposure in MCF-7 cells.

Overall, these results indicate that doxorubicin induced NF-κB nuclear translocation in all tested breast cancer cells, but they showed a differential NF-κB transcriptional response probably refecting their differences at the genetic background.

### Doxorubicin enhances the transcription of NF-κB regulated gene products in breast tumors ex vivo

**Figure 3:**
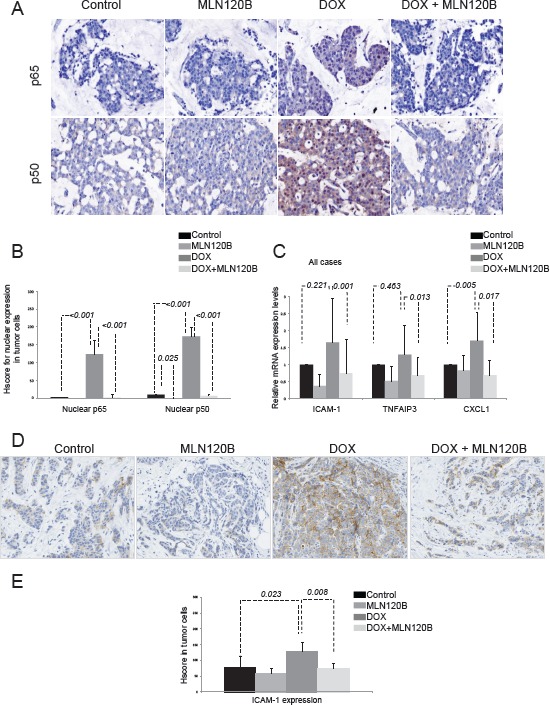
Doxorubicin induces p65/p50 nuclear translocation *ex vivo* in breast tumors and increases NF-κB-driven transcription A set of 20 human breast tumors were exposed *ex vivo* to vehicle, 20 µM MLN120B, 2 µg/ml doxorubicin or both during 24h and then formalin-fixed paraffin embedded (FFPE) tissues were prepared. A, Representative immunohistochemical images of FFPE sections control and doxorubicin treated stained with p65 and p50. B, Graph showing the IHC HScores for nuclear p65 and p50 stainining in all samples relative to control condition. C, Total RNA from *ex vivo* human breast tumors was extracted for analysis of the *ICAM-1* and *TNFAIP3* and *CXCL-1* genes by qRT-PCR. The relative target gene expression level was also normalized to the *RPLP0* expression in each sample. D, Representative immunohistochemical results of ICAM-1 staining in sections of the FFPE breast specimens; control (left panel), doxorubicin 5µM 24h (middle panel) and 20µM MLN120B plus doxorubicin 5µM 24 hours (right panel). E, Graph show the average of ICAM-1 expression determined by immunohistochemistry in all samples relative to control condition.

We next assayed whether the effects of doxorubicin on NF-κB that we observed *in vitro* also occurred in human breast cancer. For that, we exposed a panel of 20 freshly isolated human tumors *ex vivo* to doxorubicin and MLN120B alone or combined [17]. In control conditions, p65 and p50 were mainly detected in the cytoplasm. After doxorubicin treatment, both NF-κB proteins translocated to the nucleus of tumor cells. Moreover, doxorubicin-induced nuclear accumulation of p65 and p50 subunits was efficiently prevented by MLN120B (Fig. 3A and 3B). These findings were corroborated in an independent series of 36 breast tumors, which were treated *ex vivo* with doxorubicin (data not shown).

Next, we evaluated whether p65 and p50 nuclear translocation in doxorubicin-treated tumor specimens was associated with an increase in the expression of NF-κB target genes *TNFAIP3, ICAM-1* and *CXCL-1. IL8* gene was not included for analysis because of very low expression detected in breast cancer specimens. RNA for gene expression data was obtained in 13 samples. Doxorubicin induced the transcriptional upregulation of *ICAM-1*, in 61.5% of cases, of *TNFAIP3* in 46.2% of cases, and of *CXCL1* in 75% of cases, compared with control conditions (Supplementary Fig. S2B). This effect was strongly counteracted with MLN120B, which produced a significant downregulation of *ICAM-1, TNFAIP3* and CXCL-1 expression (p=0.001, 0.013 and 0.017, respectively) (Fig. 3C). Similarly, we found that doxorubicin treatment *ex vivo* significantly increased levels of *ICAM-1* in the cytoplasm and membrane of tumor cells (p=0.023), which was prevented by MLN120B (p=0.008) (Fig. 3D and 3E).

### Deficiency in tumor suppressor p53 is required for doxorubicin induced transcriptional upregulation of NF-κB target genes

**Figure 4:**
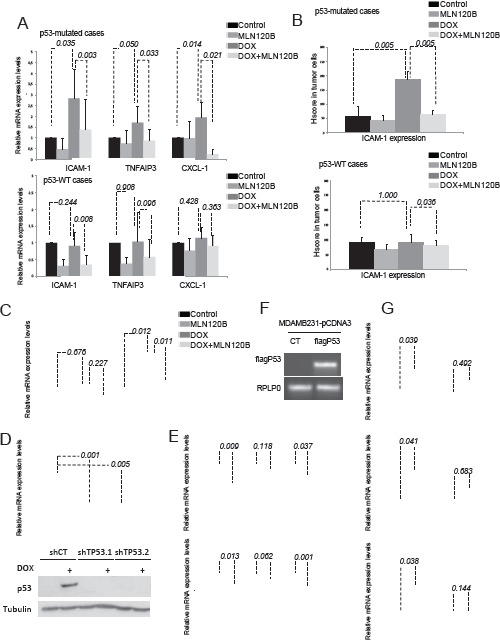
p53 deficiency is required for NF-κB-driven transcription induced by doxorubicin A, B, graph shows the relative levels of *ICAM-1*, *TNFAIP-3* and *CXCL-1* obtained by qRT-PCR (a) and ICAM-1 determined by IHC (b) taking into account all tumors in which p53 has been considered as WT or deficient status by immunohistochemistry. C, MEFs WT and p53-/- were preincubated 1h 30 min with MLN120B 205µM and exposed to 5µM doxorubicin for 4hours. qRT-PCR analysis of *ICAM-1* was performed and normalized to the *RPLP0* expression in each condition. p53 expression in MEFs was determined by WB, tubulin expression was used as loading control. D, MCF-7 were infected with 3 different lentiviral particles coding for; shCT and 2 shRNA against TP53 (shTP53.1 and shTP53.2); qRT-PCR of TP53 relative to RPLP0 and Western Blot determination of p53 in cell lysates from cells exposed to 5µM doxorubicin for 24h was were used as knocking down controls. E, MCF-7 shCT and shTP53 were exposed for 4hours to doxorubicin 5µM. Relative expression levels of ICAM-1 and TNFAIP3 were determined by qRT-PCR in each condition. RPLP0 expression was used for normalization. F, MDA-MB-231 were transfected with pCDNA3CT vector and pCDNA3fagp53. RNA was extracted from both transfected cell lines and RT-PCR was done to detect fag p53 expression. RPLP0 was used as loading control. G, qRT-PCR analysis of *ICAM-1*, *CXCL-1* and *TNFAIP3* was performed in MDA-MB-231pcDNA3CT and pCDNA3 fagp53 after 4 hours of 5µM doxorubicin treatment. The relative target gene expression level was also normalized to the *RPLP0* expression in each condition. The graph shows the results of the expression of the different genes in each condition relative to their expression in control condition. Mean and SE from triplicate experiments are indicated.

A crosstalk between NF-κB and p53 transcription factors, which are both activated by DNA damage exists [18, 19]. Thus, we hypothesized that the different p53 status of specific breast cancer cells could explain differences on NF-κB transcriptional activation by doxorubicin. Supporting this idea, among the cells analyzed, only in MCF-7 cells that carry wild-type p53, doxorubicin-induced nuclear NF-κB translocation did not result in activation of target genes. On the contrary, in the mutant p53 MDA-MB-231, SKBR3 and BT-474 cells, NF-κB target genes were activated by doxorubicin. So, we hypothesized that p53 status may be associated with different NF-κB responses upon doxorubicin treatment. In ex vivo experiments using fresh human breast cancer, p53 mutation was indirectly assayed by its nuclear accumulation in tumor cells by IHC [20]. We found that NF-κB target gene activation by doxorubicin was significantly associated with p53 nuclear accumulation (Fig. 4a). Specifically, expression of tested NF-κB target genes was significantly increased by doxorubicin in all p53 deficient tumors (n=5) (p=0.035 for *ICAM-1*, p=0.050 for *TNFAIP3* and p=0.014 for CXCL-1), but not in p53 wild type tumors (n=8) (p=0.244 for *ICAM-1*, p=0.908 for *TNFAIP3* and p=0.428 for CXCL-1) (Supplementary Fig. S2A). These results were confirmed in an independent set of samples that were treated with doxorubicin alone (Supplementary Fig. S2B). *ICAM-1* expression was significantly increased by doxorubicin in p53-defcient tumors (p=0.005) and this increase was prevented by MLN120B (p=0.005), whereas this effect was not observed in p53 wild type cases (p=1.000) (Fig. 4B).

Interestingly, p53-dependance in NF-κB target gene expression induced by doxorubicin was also observed in WT and p53KO MEFs (Fig. 4C). We found that p53KO but not WT MEFs significantly increased *ICAM-1* mRNA levels through NF-κB activation in response to doxorubicin treatment (p=0.012 and p=0.676, respectively). Importantly, MLN120B induced downregulation of *ICAM-1* only in p53KO cells (p=0.011).

To confirm that p53 was the responsible of impairing NF-κB transcriptional activity induced by doxorubicin in breast, we knocked down p53 expression using two different shRNA in MCF-7 cells (Fig. 4D). Both shRNA (shTP53.1 and shTP53.2) decreased p53 mRNA expression and avoided an efficient induced expression of the p53 protein after doxorubicin treatment, as observed in the cells expressing the shRNA control (shCT). Unexpectedly, no differences were detected in *ICAM-1* and *TNFAIP3* mRNA levels between CT and p53 knocked down cells when treated with doxorubicin (Fig. 4E). On the contrary, overexpression of p53WT in the p53 mutant cells MDA-MB-231 using pCDNA3fagp53 (Fig. 4F) prevented doxorubicin-mediated induction of NF-κB target genes (Fig. 4G), and effect was maintained in cells transfected with the control vector.

These results confirm that p53 null status seems to be necessary for doxorubicin to activate NF-κB -dependent transcription in breast cancer cells both *in vitro* and *ex vivo*, although other factors might be limiting NF-κB response to DNA damage.

### Nuclear p65 expression and p53 deficiency are associated with poor prognosis in breast cancer patients

Finally, we tested whether the presence of nuclear p65 and nuclear p53, as a surrogate of p53 inactivation, in breast tumors predicted differences in outcome. In a cohort of 335 early breast cancer patients, nuclear accumulation of p53 was observed in 85 (25.4%) patients, and nuclear p65 in 118 (35.2%) of cases (Supplementary Table S2).

Detection of nuclear p65 significantly only correlated with relapse of the disease (30.5% of cases with nuclear p65 vs. 15.2% without expression of nuclear p65; p=0.001). Nuclear p53 was associated with high tumor size (p=0.011), high tumor grade (52.9% in cases with nuclear p53 vs. 31.0% in p53 negative tumors; p=0.001), absence of ER (65.9% vs. 13.9%; p<0.001), HER2 amplification (31.8% vs. 15.5%; p=0.001), high proliferation (35.3% vs. 22.5%) and relapse of the disease (31.8% vs. 16.8%; p=0.003).

In addition, detection of nuclear p65 significantly correlated with the nuclear accumulation of p53 in these tumors (p=0.003). Nuclear p65 and p53 co-localization was found in 42 (12.5%) of the studied patients and was associated with high tumor grade (52.4% in cases with co-localization vs 34.2% in cases without co-localization; p=0.030), negativity for ER (64.3% vs 21.7%; p=0.001), HER2 amplification (35.7% vs 17.3%; p=0.005), high proliferation (38.1% vs 24.0%; p=0.042) and relapse (38.1% vs 18.1%; p=0.003).

**Figure 5:**
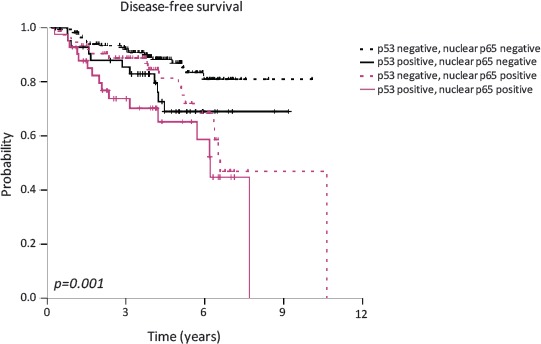
Association between p53 and p65 nuclear localization and disease-free survival in breast cancer patients High-risk patients were stratified according to high or low p53 nuclear accumulation status and high or low nuclear NF-κB p65 staining.

Finally, DFS analysis showed a higher risk of relapse in breast cancer patients with nuclear accumulation of p53 and p65 in tumor cells (p=0.001). The hazard ratio for relapse in patients with nuclear p65 and p53 tumors was 2.49 (IC 95%; 1.42-4.37) (Fig. 5) (Table 1). Kaplan-Meier curves for relapse and log-rank test comparisons also showed that tumor size (p=0.001), grade (p=0.022), axillary lymph node involvement (p=0.001), ER and/ or PR expression (p=0.011), HER2 amplification (p=0.014), triple negative phenotype (p=0.036), adjuvant hormonotherapy (p=0.025), p53 nuclear expression (p=0.004) and p65 nuclear expression (p=0.002) were associated with the risk of relapse (Table 1).

**Table 1:** Uni- and multivariate Cox analysis for disease-free survival in series of breast cancer patients (n=335), including clinico-patological factors and p65 and p53 53 nuclear expression

		Univariate (n=335)			Multivariate (n=335)		
Variable		HR	95% CI	P	HR	95% CI	P
Age				0.214			-
	Premenopausal	1.00			-		
	Postmenopausal	1.40	0.82 to 2.38		-		
Tumor size, mm				0.001			0.115
	≤20	1.00			1.00		
	21-50	2.21	1.31 to 3.72		1.96	1.02 to 3.75	
	>50	2.82	1.32 to 6.03		1.98	1.04 to 6.11	
Tumor grade				0.022			0.521
	I	1.00			1.00		
	II	1.25	0.36 to 1.91		0.98	0.55 to 2.16	
	III	1.74	0.84 to 3.76		1.12	0.40 to 3.13	
Lymph nodes				0.001			0.048
	None	1.00			1.00		
	1-3	1.06	0.59 to 1.89		1.10	0.55 to 2.20	
	4-9	1.75	0.83 to 3.70		1.11	0.44 to 2.79	
	>9	4.27	2.02 to 8.74		3.69	1.32 to 10.02	
Histology				0.858			-
	Ductal	1.00			-		
	Lobular	0.96	0.85 to 1.15		-		
	Others	0.96	0.16 to 3.52		-		
Hormonal receptor status				0.011			0.656
	Negative	1.00			1.00		
	Positive	0.53	0.32 to 0.86		1.13	0.37 to 4.77	
HER2 status				0.014			0.369
	Negative	1.00			1.00		
	Positive	1.89	1.14 to 3.14		1.53	0.61 to 3.89	
Triple negative phenotype				0.036			0.354
	No	1.00			1.00		
	Ye	1.76	1.04 to 2.98		1.89	0.49 to 7.31	
Proliferation (Ki-67)				0.089			-
	Low proliferation (<14%)	1.00			-		
	High proliferation (≥14%)	1.31	0.75 to 5.26		-		
Adjuvant chemotherapy				0.416			-
	No	1.00			-		
	Ye s	0.84	0.41 to 1.71		-		
Adjuvant hormonotherapy				0.025			0.216
	No	1.00			1.00		
	Yes	0.53	0.31 to 0.49		0.63	0.31 to 1.29	
p53				0.004			0.511
	Negative	1.00			1.00		
	Positive	2.03	1.25 to 3.31		1.37	0.53 to 3.58	
Nuclear p65				0.002			0.329
	Non-overexpression	1.00			1.00		
	Overexpression	2.11	1.31 to 3.40		1.56	0.48 to 1.76	
p53 and p65 nuclear co-expression				0.001			0.009
	No	1.00			1.00		
	Ye s	2.49	1.42 to 4.37		2.64	1.27 to 5.49	

Abreviations: DFS, free survival; HR, hazard ratio; CI, confidence interv al; HER2, human epiderma l growth factor re ceptor 2

A multivariate analysis was performed including all the significant clinico-pathological factors (Table 1). Nuclear co-localization of p65 and p53 retained its adverse prognostic role for relapse (P<0.009). The hazard ratio for relapse in patients with tumors with accumulation of nuclear p65 and p53 was 2.64 (IC 95%; 1.27-5.49). Another independent prognostic factor for DFS was lymph node involvement (p=0.048).

These results indicate that the p53 status in breast cancer tumors predicts the NF-κB -dependent transcription of genes in response to doxorubicin.

## DISCUSSION

The DNA-binding activity and transcriptional activation of NF-κB after doxorubicin treatment is still under debate [16, 21]. In this work we show that doxorubicin induces, through NF-κB activation, a transcriptome compatible with metastasis and chemoresistance exclusively in a p53-defcient cell context. Moreover, deficient p53 status and nuclear NF-κB correlated with worse outcome of breast cancer patients. Doxorubicin is able to promote NF-κB translocation, DNA binding and upregulation of NF-κB dependent transcription leading to expression of a NF-κB-dependent transcriptome associated with tumor growth, migration, metastasis and chemoresistance in mutant p53 MDA-MB231, BT-474 and SKBR3 cells, but not in wild-type p53 MCF-7 cancer cells. The correlation between p53 status and the ability of doxorubicin to elicit a tumorigenic and anti-apoptotic NF-κB response was also observed in a large panel of fresh human breast tumors ex vivo treated with doxorubicin. In addition, our studies using genetic manipulation have demonstrated the direct role of p53 in the NF-κB response to doxorubicin.

After DNA damage, the sequential molecular events that culminate in the p65/p50 nuclear localization and DNA-binding activity of NF-κB are widely accepted [3, 22]. However, it remains uncertain whether the NF-κB activated by doxorubicin and other related chemotherapeutic agents elicit a transcriptomic response. Several studies have shown that after causing DNA-binding, doxorubicin will induce NF-κB transcriptional events and lead to the production of antiapoptotic gene products that can contribute to chemoresistance [23]. Indeed, doxorubicin can stimulate the expression of prosurvival NF-κB genes, like *BCLl-XL*, a member of the Bcl-2 family in cancer cells [24]. In breast cancer, NF-κB has been assigned as a pro-apoptotic pathway through repression of anti-apoptotic genes in response to doxorubicin [25], and in other reports, doxorubicin-induced nuclear p65 cannot be phosphorylated and acetylated and, therefore, its DNA binding affinity and transcriptional activity is reduced [16]. Anthracycline-induced DNA-binding activity of NF-κB in osteosarcoma has been dually described as activator and repressor of antiapoptotic genes. It is proposed that DNA-intercalation agents would activate a repressive NF-κB complex while those that do not intercalate DNA would induce an active NF-κB complex [26]. Therefore, most of the studies conclude that NF-κB activated by DNA damage may activate or repress genes depending on tumor type, context and environmental conditions. In fact, the Ser536 phosphorylation of p65 has been proposed as a marker for transcriptionally activation or repression of NF-κB by doxorubicin, respectively [21]. In our work, we found that NF-κB induced the expression of *CXCL1, ICAM1* and *TNFAIP3* after doxorubicin treatment only in a subset of tumors, suggesting that differences in responses may exist among tumor subtypes.

We selected different genes to confirm our microarray data based on the relevance in chemoresistance and metastatasis. The TNFAIP3 gene, also known as A20, is a deubiquitinating enzyme and a negative regulator of NF-κB [27]. Its overexpression is associated to aggressiveness of nasopharyngeal [28] and breast [29] cancer and also with the development of resistance to O6-alkylating agents in glioblastoma [30], and tamoxifen resistance [29]. ICAM-1 is a surface transmembrane glycoprotein belonging to the immunoglobulin superfamily of adhesion molecules and plays a role in immune response, but also is involved in mechanisms of invasion and metastasis in breast cancer [31]. *ICAM-1* expression has been correlated with progression, prognosis and aggressive phenotype in cancer [32, 33]. Moreover, and in agreement with our results, increased plasma levels were observed in doxorubicin-treated breast cancer patients [34]. The other selected gene was the chemokine CXCL-1, which promotes chemotaxis in different cell types such as stromal-epithelial and host-tumor cells, and consistently, is involved in metastasis, tumor progression and chemotherapy resistance and survival [35]. *In vivo* downmodulation of CXCL-1 reduces the metastatic potential in MDA-MB-231 and enhanced anti-tumor effects of chemotherapy [36]. Thus, according to these data and our own results, doxorubicin-induced NF-κB activation in a p53 defcient background might favor the acquisition of a metastatic phenotype of breast cancer, and residual cells after treatment would be more aggressive resulting in tumor promotion to dissemination.

DNA damage induces stabilization of tumor suppressor p53 and could eventually lead to cell cycle arrest, senescence or apoptosis [37]. An antagonistic crosstalk between p53 and NF-κB networks has been described because directly compete for binding to the transcriptional co-activator CBP/p300 by regulation of IKKκ phosphorylation [38]. According to that, it has been shown that NF-κB inhibition by curcumin enhances chemotherapy efficacy by favoring induction of p53-p300 dependent apoptosis in breast cancer [39].

Our results showed that mutated p53 mediated the induction of transcription of NF-κB regulated genes by doxorubicin and functional restoration of wild-type p53 impaired doxorubicin- NF-κB dependent transcription in breast cancer. Similarly, mutant p53 increases NF-κB transcriptional activity induced by TNFα and knocking down mutant p53 reduced NF-κB activity. However, when we knocked down wild type p53 in the MCF-7 cells, NF-κB target gene expression upon doxorubicin treatment did not change. This observation suggests that other factors play a role in chemotherapy responses and gene regulation. Indeed, the ER, which is expressed in MCF-7 and in two-thirds of breast cancers [40], has also been associated with NF-κB activity. Breast cancer cells expressing ER contain lower NF-κB activity than ER-negative cells [41]. Moreover, ectopic expression of the receptor in ER-negative cells reduced NF-κB DNA binding activity and expression of several NF-κB target genes [42]. Thus, despite the requirement for further functional studies combining both ER and p53 effects on NF-κB activity, the ER status also determines the NF-κB activity independently of p53.

In our series, nuclear accumulation of p53 protein, as a marker of p53 deficiency [20], was associated with an increase of NF-κB target genes in response to doxorubicin ex vivo. p53 deficiency appears to be an independent marker of poor prognosis and may influence chemotherapy response in breast cancer [39]. A significant association of co-expression of p53 and nuclear p65 with worse outcome was found in our study. According to our hypothesis, and considering that in multivariate analysis the nuclear colocalization of p53 and p65, but not of each transcription factor alone, retained its prognostic value, our results strongly suggest that p53 mutant breast tumors are more likely to relapse earlier to chemotherapy because of the ability to activate NF-κB target genes. Furthermore, those p53-defcient tumors with active NF-κB before treatment will have fewer chances to benefit from therapy. In another study of colorectal cancer, it has been observed that loss of p53 during tumor progression is associated with an NF-kB-dependent inflammatory microenvironment and the induction of epithelial-mesenchymal transition [43]. In this study, NF-kB activation in p53 deficient tumors was caused by bacterial products from the colonic mucosa, and a role of NF-κB in the progression to metastasis was clearly demonstrated. Also in head and neck lesions, nuclear p65 and a novel NF-κB gene signature correlated with mutant p53 status [44]. Also related to NF-κB and p53 relationship, a recent publication shows that mutant p53 prolongs NF-κB activation in colorectal cancer cells [19].

In summary, we show that doxorubicin-driven NF-κB transcription occurs in p53-defcient breast tumors and specifically the expression of metastasis and chemoresistance-associated genes. This NF-κB response occurs in a high percentage of breast tumors, therefore anthracyclines-based regimens in p53-defcient breast tumors should be revisited. Based on our and other results, incorporation of NF-κB inhibitors, such as small molecules targeting the IKK complex or other indirect NF-κB inhibitors (i.e. PARP inhibitors), should be considered to avoid the expression of genes involved in chemoresistance and relapse and, thus improving the clinical benefit and the outcome of breast cancer patients.

## METHODS

### Cell lines and reagents

MDA-MB-231, BT-474, MCF-7 and SK-BR-3 human breast cancer cell lines were obtained from American Type Culture Collection (ATCC) and cultured at 37°C in 5% CO2. Wild-type murine embryonic fibroblast (WT MEFs) and p53KO-MEFs were provided by Dr. Yelamos (IMIM, Barcelona). All cells except MCF-7 were cultured in DMEM/F12 supplemented with 2mM L-glutamine, penicillin (100units/ml), streptomycin (100μg/ml) and 10% FBS. MCF-7 were grown in DMEM with same supplements listed above. For BT-474, insulin (0.01µg/ml) was added. Cells obtained from the ATCC were tested via STR analysis by ATCC experts at the end of experimental work. Doxorubicin (Sigma-Aldrich, St. Louis, MO) was dissolved in deionized water at 10mM. MLN120B was provided by Millennium Pharmaceuticals (Cambridge, MA) and stored as a 10mM stock in DMSO at -20°C.

### Immunofluorescence

Cells grown in 12-well plate preloaded with sterile coverslips were treated, washed and fixed. Slides were incubated for 1h at room temperature with primary antibody (p65, sc-372) and incubated with Alexa 488-coupled secondary antibodies (Life Technologies, Grand Island, NY) for 30min. Nuclei were counterstained with 4,6-diamidino-2-phenylindole (DAPI, Abbott Molecular, Abbott Park, IL). Cells were visualized under fluorescence microscopy.

Electrophoretic mobility shift assay (EMSA)

Nuclear cell extracts were obtained as described [45]. EMSAs were carried out with a specific NF-κB consensus probe end-labeled with [γ-32P] ATP by incubation with T4 polynucleotide kinase at 37°C for 1h and purified in a G-50 spin column (Amersham Biosciences, Piscataway, NJ). Extracts were incubated with 1µL of the 32P-labeled probe and binding buffer for 15min. DNA-protein complexes were separated from unbound oligonucleotides in 8% polyacrylamide gels using Tris/borate/EDTA. The specificity of DNA-protein complexes were confirmed by competition with 100-fold excess of unlabeled NF-κB-probe. After electrophoresis, gels were fixed and then dried and exposed to X-ray film at -80°C.

### Western Blot

Western blot was performed as previously described [46], using anti-IκBα, anti-*ICAM-1* and anti-p53 antibodies (Cell Signaling, Danvers, MA), anti- NF-κB p65 and p50 (Santa Cruz Biotech, Dallas, TX) and anti-α-tubulin antibody (Sigma-Aldrich) as loading control.

### NF-κB DNA-binding ELISA-based assay

Nuclear extracts were obtained using the Nuclear Extract Kit (Active Motif, Carlsbad, CA) DNA binding activity of NF-κB was measured by an ELISA-based assay using a TransAM NF-κB kit (Active Motif) following manufacturer's instructions. This assay detects binding of p65, p50, p52, c-rel and Rel-B proteins to oligonucleotides containing an NF-κB consensus-binding site immobilized onto 96-well plates by specific primary antibodies that recognized an epitope that is accessible only when NF-κB is activated and bound to its target DNA, and was quantified by spectrophotometry.

### Luciferase reporter assay

MDA-MB-231 cells were transfected with a plasmid encoding a luciferase reporter gene under a promoter containing repeats consensus of NF-κB binding sites [45]. Cells (6x105 cells/well) were seeded in 12-well plates and allowed to attach overnight. After serum starvation for 24h, cells were incubated during 1.5h with 20µM MLN120B and then with doxorubicin 5µM for 24h. Cells were lysed and luciferase activity was measured using the Dual-LuciferaseTM Reporter Assay System (Promega, Madison, WI) following the manufacturer's instructions in 96-well plates by a luminometric reader (Thermo Scientific, Nunc Brand, Lafayette, CO).

### RNA extraction for gene expression studiesα

Total RNA from cell lines was isolated using RNeasy mini Kit (Qiagen, GMBH). Formalin-fixed paraffin-embedded (FFPE) breast tumor samples treated ex vivo were subjected to standard deparaffinization prior to and RNA extraction using RNeasy FFPE kit (Qiagen), including an overnight proteinase K treatment and RNase-free DNase I processing for 30min. RNA purity and integrity were assessed both by spectrophotometry (NanoDrop ND-1000, NanoDrop Technologies, Wilmington, DE) and electrophoresis (2100 Bioanalyzer, Agilent Technologies, Santa Clara CA) considering for microarray experiments RNA purity, A260/280>2.0 and A260/230>1.4 and RIN>9.4, as minimal requirements.

### Microarray analysis

Microarray expression profiles were obtained using the Affymetrix GeneChip Human Exon 1.0 ST Array (Affymetrix Inc, Santa Clara, CA). Amplification, labeling and hybridizations were performed according to protocols from Ambion (Applied Biosystems, Foster City, CA) and Affymetrix. Briefly, 200ng of total RNA were amplified using the Ambion WT Expression Kit (Applied Biosystems), labeled using the WT Terminal Labeling Kit (Affymetrix), and then hybridized to Human Exon 1.0 ST Array for 16h at 45°C in a GeneChip Hybridization Oven 640. Following hybridization, array was stained in the Affymetrix GeneChip Fluidics Station 450 and scanned using a GeneChip Scanner 3000 7G.

### Gene expression profile analysis

After quality control of raw data, it was background corrected, quantile-normalized and summarized to a gene-level using the robust multi-chip average (RMA) obtaining a total of 18708 transcript clusters. Normalized data was then filtered to avoid noise created by non-expressed transcript clusters in the condition. Only transcripts with an intensity signal of more than a 10% of all intensities of the mean of studied groups and then over 50% of variance from total resting variance were considered for further analysis, which lead to 8535 transcript clusters. Linear Models for Microarray (LIMMA), a moderated t-statistics model, was used for detecting differentially expressed genes between conditions. Correction for multiple comparisons was performed using false discovery rate and only genes with an adjusted p-value <0.05 were selected as significant. For functional analysis purposes, genes were selected to have a non-adjusted p-value <0.05. Hierarchical cluster analysis was also performed. All data analysis was performed in R v2.11.1 with packages Aroma. Affymetrix, Biobase, Affy, LIMMA and genefilter. Functional analysis was performed with Ingenuity Pathway Analysis v9 (Ingenuity Systems, Redwood City, CA).

### Real-Time Quantitative PCR (RT-qPCR)

Primers were designed with the DNAstar Primer design software (DNASTAR, Inc, Madison, WI) and the NCBI database. Specific primers for IL-8 (NM_000584.3) Fw: 5'- GACAGCAGAGCACACAAGC-3'; Rv: 5'- GGCAAAACT GCACCTTCAC-3'; *TNFAIP3*(NM_006290.2) Fw: 5'- GGACTCCAGAAAACAA GGGC-3'; Rv: 5'- CTGGAACCTGGACGCTGTG-3'; CXCL-1 (NM_001511.2) Fw: 5'- GAAAGCTTGCCTCAATCCTG-3'; Rv: 5'- CAGGAACAGCCACCAG TGAG- 3'; *ICAM-1 *(NM_000201.2) Fw: 5'- GGCAGTCAACAGCTAAAACC-3'; Rv: 5'- GCGTAGGGTAAGGTTCTTGC-3'; RPLP0 (NM_001002.3) Fw: 5'-GCAGGTGTTCGACAATGGC-3'; Rv: 5'-CTGGCAACATTGCGGACAC-3'; TP53 Fw: 5'-GCCCCTGTCATCTTCTGTC-3'; Rv: 5'-GGGAGTACGTGCAA GTCAC-3'; FLAGp53 Fw: 5'-CATGGACTACAAGGACGAC-3'; Rv: 5'-CAGGAAGTAGTTTCCATAGG-3'. Specific probes from Universal probe library (Roche Applied Science, Mannheim, GE) were. RPLP0 was used as housekeeping gene. RNA was reversely transcribed to cDNA using High Capacity cDNA Reverse Transcription kit (Applied Biosystems). For the *ex vivo* samples, 40ng of cDNA synthesized were preamplificated using TaqMan PreAmp Master Mix (Applied Biosystems). DNA amplification was done in a Lightcycler 480 RT PCR-System at 45 cycles. Relative gene expression was calculated according to the comparative cycle threshold (Ct) method.

### Transfection and infection

The vector pLKO1.puro was used for shRNA knock down of p53 expression using two independent shRNA sequences (TRCN0000003756, TRCN0000342259, Sigma-Aldrich). HEK293T cells were transfected using PEI reagent. After 24h, supernatant with lentiviral particles was used to infect MCF-7 cells. Two days after infection puromycin (2µg/ml) was added and surviving cells were pooled to generate stable cell lines. pCDNA3fagp53 and CT vector were purchased from Addgene (Cambridge, MA) and were transfiected into MDA-MB-231 cells using lipofectAMINE 2000 (Life Sciences). Neomycin was added to select transfected cells. Same protocol was used to transfect the NF-κB luciferase reported plasmid [45].

### Human samples

Breast tissues were surgical resection specimens from primary tumors obtained from Biobanks of Parc de Salut Mar (MARBiobanc, Barcelona), Fundacion Jimenez Diaz (BFJD, Madrid) and Valencia Clinic Hospital. FFPE tumor specimens (N=126) were retrospectively selected from consecutive breast cancers patients diagnosed between 1998-2000, which had fulfilled the following criteria; infiltrating carcinomas, operable disease, no neoadjuvant therapy, enough available tissue and clinical follow-up. Clinical data were collected from medical clinical records by oncologists. TNM (Tumor, Node, Metastasis) staging was classified using the American Joint Committee on Cancer (AJCC) staging system for breast cancer. Histological grade was defined according Scarff-Bloom-Richardson modified by Elston criteria [47]. Estrogen (ER) and progesterone receptor (PR) expression was determined by immunohistochemistry (IHC) (SP1 and PgR636 clones, respectively, Dako, Glopstrup, DK), establishing positivity criteria in ≥1% of nuclear tumor staining, following ASCO/CAP guidelines [48]. HER2 amplification was assayed by Fluorescence in Situ Hybridization (FISH) (Pathvysion, Abbott Molecular), following ASCO/CAP recommendations [49]. Proliferation marker Ki-67 was studied by IHC (MIB1 clone, Dako) and percentage of stained cells was scored [50]. P53 expression was assayed by IHC (clone DO-7, Dako). The study was approved by the Ethics Committees of the three institutions. Tissue microarrays (TMA) were constructed from representative areas of each tumor including three 1mm tissue cores using a TMA workstation (T1000 Chemicon) [46].

Additional 20 breast tumors, which were not needed for diagnostic purposes, were obtained from surgical specimens of patients newly diagnosed for invasive cancer for the human breast tumor *ex vivo* models. Samples were processed in sterile conditions immediately according to our experience [46]. One slice (control sample) was selected and additional slices (treated samples) were exposed to doxorubicin alone at 2μg/ml; MLN120B at 20µM alone and doxorubicin plus MLN120B for 24h at 37°C, 5% CO2. Specimens were fxed in 10% neutral-buffered formalin for 24hs.

### Immunohistochemistry

Tissue sections (3μm) placed on plus charged glass slides. After deparaffnization, heat antigen retrieval was performed in pH9 EDTA-based buffer (Dako). Endogenous peroxidase was blocked and slides were incubated with primary antibodies (p65; sc-372, p50; sc-114, from SantaCruz; *ICAM-1* from Cell Signaling) for 60min, followed of appropriate anti-Ig horseradish peroxidase-conjugated polymer (Flex+, Dako) using a Dako Autostainer, and visualized with 3,3'-diaminobenzidine. Sections incubated with non-immunized serum were used as negative controls. As positive control, breast tumors with a known expression of markers were used.

### Statistical analysis

Statistical analysis was carried out with SPSS version 13.0 (SPSS, Inc, Chicago, IL). To analyze correlations between p65 and p50 expression and clinico-pathological variables we used χ2 test (Fisher exact test), based on bimodal distribution of data. Disease free survival (DFS) was considered from date of surgery to date of any primary, regional or distant recurrence of the infiltrating carcinoma, as well as appearance of a secondary tumor or death. Univariate analysis for DFS curves were constructed based on Kaplan-Meier life-table method and analyzed using log-rank test of equality across strata; all predictors with P values lower than 0.05 were used in multivariate analysis using the Cox proportional hazards model. Analysis of experimental conditions was done by paired T test. All the statistical tests were conducted at two sided 0.05 level of significance. This work was performed in accordance with Reporting Recommendations for Tumor Marker Prognostic Studies (REMARK) guidelines [51].

## Supplementary Figures and Tables




