# Supplementary material for: Deficiency in p53 is required for doxorubicin induced transcriptional activation of NF-κB target genes in human breast cancer
Source: Oncotarget. 2013 Nov 23;5(1):196–210. doi: 10.18632/oncotarget.1556 (PMC3960201; doi:10.18632/oncotarget.1556)
Supplement: Supplementary file 1 [file oncotarget-05-0196-s001.pdf]

Deficiency in p53 is required for doxorubicin induced transcriptional activation of NF-κB target genes in human breast cancer - Dalmases et al

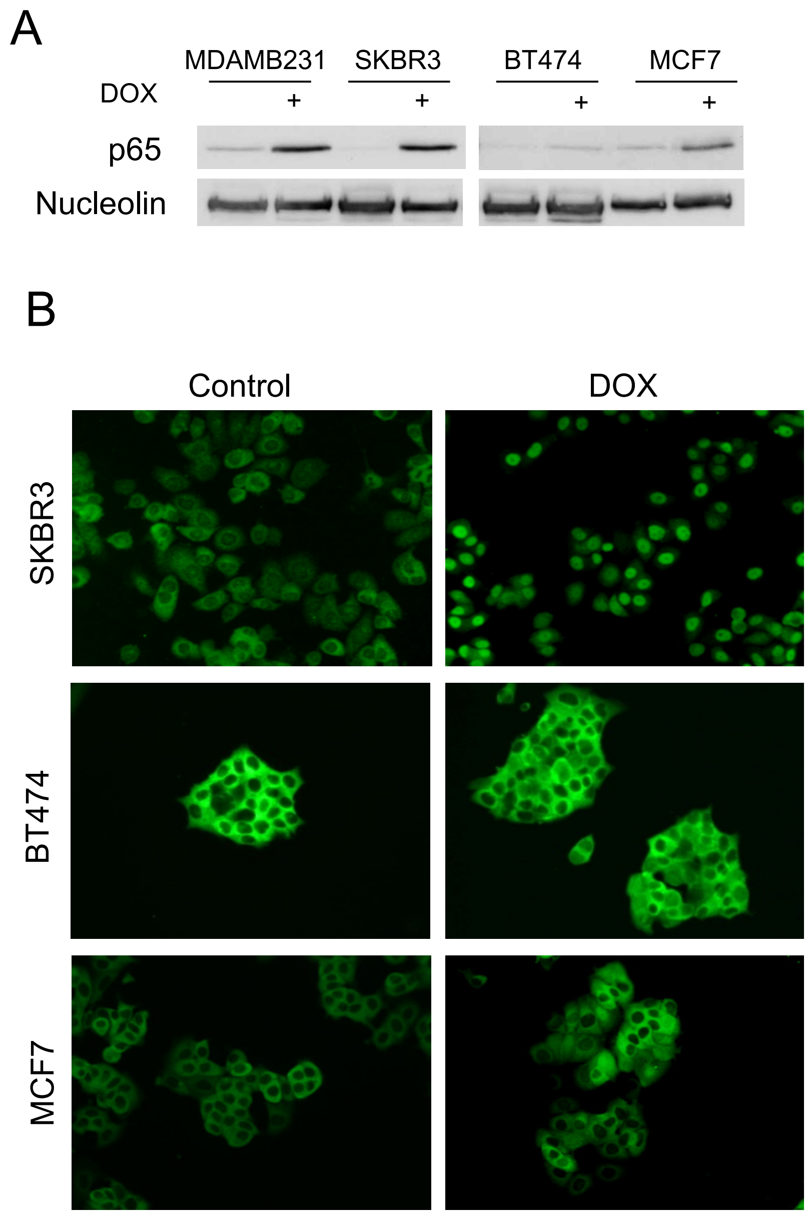

Supplementary Figure S1. Doxorubicin induces p65 nuclear translocation. MDA-MB-231, SKBR3, BT-474 and MCF-7 were exposed to 5µM doxorubicin for 4hours. A, Nuclear extracts of each condition were subjected to Western Blot analysis of p65. Nucleolin served as internal loading controls for nuclear protein. B, Cell were fixed as described in material and methods and p65 cellular distribution (nucleus/cytoplasm) was determined by immunofluorescence (IF) using Alexa 488-coupled goat anti-rabbit IgG (green).

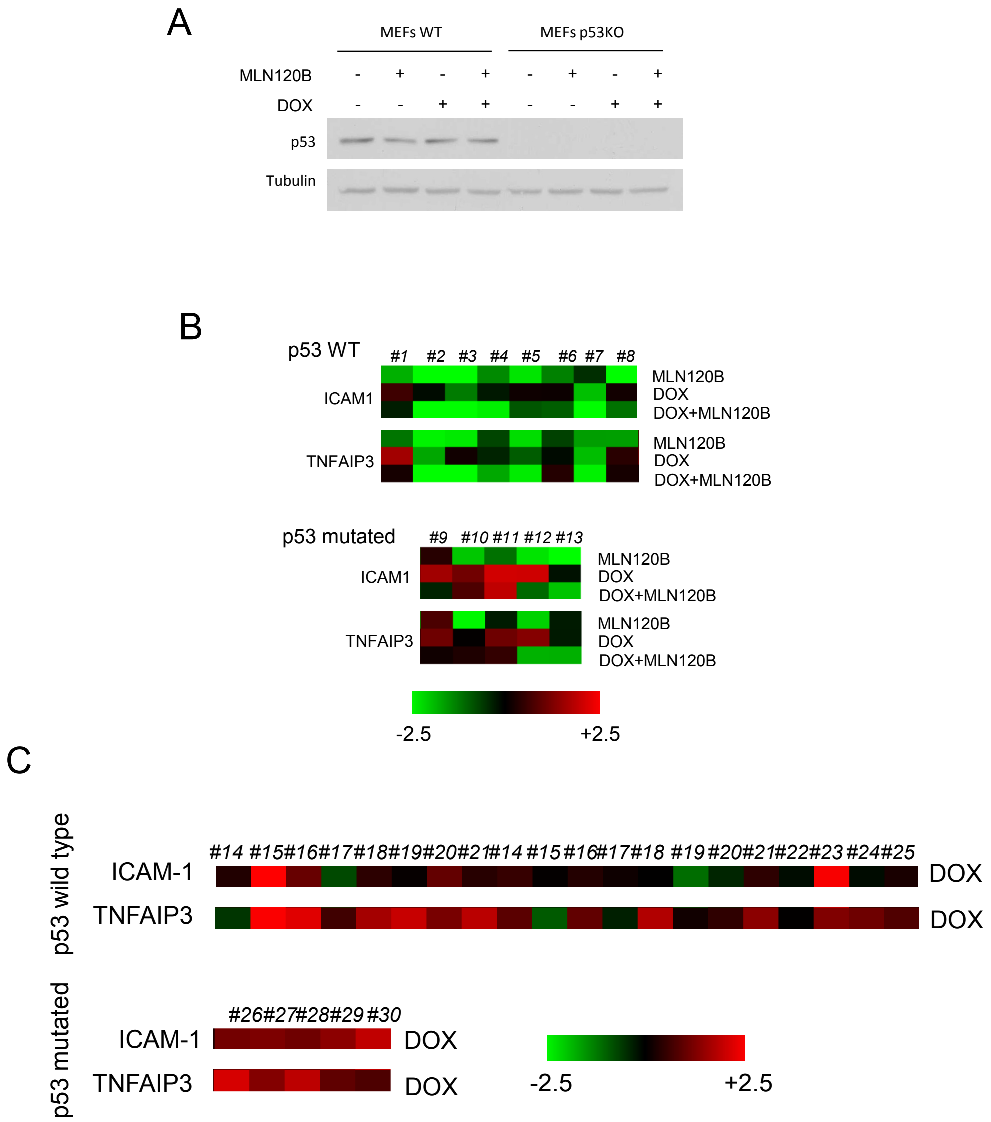

Supplementary Figure S2: NF-κB gene transcription varies depending p53 background of the breast cancer tumors. A, MEFs WT and p53-/- were preincubated 1h 30 min with MLN120B 20μM and exposed to 5μM doxorubicin for 4hours. P53 expression in MEFs was determined by WB, tubulin expression was used as loading control B, Heat maps clustering of ICAM-1 and TNFAIP3 mRNA transcript gene expression values between p53 wild type and p53 mutated cases. b. Each individual tumor exposed *ex vivo* to doxorubicin, IKKβ inhibitor and concomitantly to both drugs is represented separately. Same cases were also analyzed globally in Figure 4a. C, Heat maps clustering of ICAM-1 and TNFAIP3 mRNA transcript gene expression values in an independent series of 36 cases. Each individual tumor exposed *ex vivo* to doxorubicin is represented separately.
